# *Thrombospondin-4 *is a putative tumour-suppressor gene in colorectal cancer that exhibits age-related methylation

**DOI:** 10.1186/1471-2407-10-494

**Published:** 2010-09-16

**Authors:** Sonia A Greco, June Chia, Kelly J Inglis, Sarah-Jane Cozzi, Ingunn Ramsnes, Ronald L Buttenshaw, Kevin J Spring, Glen M Boyle, Daniel L Worthley, Barbara A Leggett, Vicki LJ Whitehall

**Affiliations:** 1 Conjoint Gastroenterology Laboratory, Royal Brisbane and Women's Hospital Research Foundation, Clinical Research Centre and Queensland Institute of Medical Research, Brisbane, Australia; 2 Immunovirology Laboratory, Queensland Institute of Medical Research, Brisbane, Australia; 3 Drug Discovery Group, Queensland Institute of Medical Research, Brisbane, Australia; 4 Royal Brisbane and Women's Hospital, Brisbane, Australia; 5 Pathology Queensland, Clinical and Statewide Services, Queensland Health, Brisbane, Australia

## Abstract

**Background:**

*Thrombospondin-4 *(*THBS4*) is a member of the extracellular calcium-binding protein family and is involved in cell adhesion and migration. The aim of this study was to evaluate the potential role of deregulation of *THBS4 *expression in colorectal carcinogenesis. Of particular interest was the possible silencing of expression by methylation of the CpG island in the gene promoter.

**Methods:**

Fifty-five sporadic colorectal tumours stratified for the CpG Island Methylator Phenotype (CIMP) were studied. Immunohistochemical staining of THBS4 protein was assessed in normal and tumour specimens. Relative levels of *THBS4 *transcript expression in matched tumours and normal mucosa were also determined by quantitative RT-PCR. Colony forming ability was examined in 8 cell lines made to overexpress THBS4. Aberrant promoter hypermethylation was investigated as a possible mechanism of gene disruption using MethyLight. Methylation was also assessed in the normal colonic tissue of 99 patients, with samples biopsied from four regions along the length of the colon.

**Results:**

*THBS4 *expression was significantly lower in tumour tissue than in matched normal tissue. Immunohistochemical examination demonstrated that THBS4 protein was generally absent from normal epithelial cells and tumours, but was occasionally expressed at low levels in the cytoplasm towards the luminal surface in vesicular structures. Forced THBS4 over-expression caused a 50-60% repression of tumour colony growth in all eight cell lines examined compared to control cell lines. Tumours exhibited significantly higher levels of methylation than matched normal mucosa, and *THBS4 *methylation correlated with the CpG island methylator phenotype. There was a trend towards decreased gene expression in tumours exhibiting high *THBS4 *methylation, but the correlation was not significant. *THBS4 *methylation was detectable in normal mucosal biopsies where it correlated with increasing patient age and negatively with the occurrence of adenomas elsewhere in the colon.

**Conclusions:**

*THBS4 *shows increased methylation in colorectal cancer, but this is not strongly associated with altered gene expression, either because methylation has not always reached a critical level or because other factors influence *THBS4 *expression. *THBS4 *may act as a tumour suppressor gene, demonstrated by its suppression of tumour colony formation *in vitro*. *THBS4 *methylation is detectable in normal colonic mucosa and its level may be a biomarker for the occurrence of adenomas and carcinoma.

## Background

Thrombospondin-4 (THBS4) is a member of the extracellular calcium-binding protein family. It is a secreted pentameric globular protein that forms part of the extracellular matrix, and functions in calcium binding, cell attachment, cell migration, cellular proliferation, cytoskeletal organisation, neurite growth, binding of other extracellular matrix components and cell to cell interactions [1-6]. *THBS4 *is expressed at high levels in the heart and skeletal muscle, is detected in neuronal tissue in the brain and eye, and also in the skin, lung, pancreas, T-cells, breast and colon tissues [1,3,5,7,8]. It is a putative tumour suppressor gene, the hypermethylation of which has been linked to cutaneous T-cell lymphoma, breast cancer and colorectal cancer (CRC) [6,8,9].

DNA methylation is the addition of a methyl group to cytosine nucleotide bases via one of the DNA methyltransferase enzymes [10]. CpG islands are most commonly found within the promoter region of genes, although they can occur in other coding and non-coding regions. More than 60% of gene promoters are found within CpG islands [11]. CpG islands are usually not methylated regardless of the expression of the gene [7]. However, if these promoter CpG islands become methylated, the associated gene may be permanently silenced, and this silencing is epigenetically inherited [10,11]. The promoter region of *THBS4 *is typical of such a CpG island that is subject to epigenetic silencing. As a putative tumour-suppressor gene, methylation of the *THBS4 *promoter and silencing of its tumour-suppressor function is potentially pathogenic.

The CpG Island Methylator Phenotype (CIMP) has been used to describe a subset of colorectal tumours with a high frequency of methylation in genes known to be specifically methylated only in neoplasia and not in normal colon [12,13]. While age-related (Type A) promoter methylation of some genes is common in both normal mucosa and neoplastic tissue, there is a subset of genes whose promoters are only found to be hypermethylated in CRC (Type C, cancer-related methylation) [12,14]. However, age-related hypermethylation in normal colonic mucosa may contribute to the pre-malignant colorectal field [14,15].

Sporadic colorectal cancer (CRC) can be divided into clinically relevant subgroups based on gene expression profiles that reflect pathways of tumour progression. The traditional pathway accounts for approximately 70% of all CRC [13]. These are characterised by chromosomal instability, gene mutations and deletions, and are microsatellite stable (MSS). Adenomas are the precursor lesions to traditional pathway colorectal cancers [16]. The serrated pathway accounts for approximately 30% of all sporadic CRC. These are characterised by a different spectrum of target gene alterations [15,17,18]. Mutations in the *BRAF *oncogene are common, along with CIMP [19]. Serrated pathway tumours have high levels of microsatellite instability (MSI-H), which results from the hypermethylation and subsequent silencing of the mismatch repair gene *MLH1*. Serrated polyps precede the development of this tumour subgroup [15].

The aims of this study were to evaluate the deregulation of *THBS4 *in a series of colorectal cancers, to examine correlations with CIMP, to probe the effect of forcing THBS4 protein expression in CRC cell lines and to examine the mechanism of *THBS4 *deregulation.

## Methods

### Patient Samples

Quantitative expression (qRTPCR) analysis was performed on 55 matched normal and tumour samples. All tumour samples collected in a fresh state were macroscopically dissected to remove contaminating normal mucosa. Quantitative methylation (qMSP) analysis was also conducted on this patient cohort as well as biopsies of normal mucosa taken during clinically indicated colonoscopy in a series of 99 patients. Biopsies were taken from each of 4 locations within the colon (caecum, transverse colon, sigmoid colon and rectum). All polyps were removed and submitted for histology. All patients gave written, informed consent, and the study was approved by the RBWH and QIMR Human Research Ethics Committees. DNA was extracted from tumour samples using a salt precipitation method as previously described [20], and from biopsy specimens using a DNA column method (Qiagen, Hilden, Germany). CIMP status was determined in tumours by normalising the methylation levels of each of the 5 Laird CIMP markers (CACNA1G, IGF2, NEUROG1, RUNX3, SOCS1) [19] by the methylation levels observed for the highly methylated *ALU *gene to generate the PMR, or Percentage of Methylated Reference. Tumours were classified as CIMP-negative (CIMP-neg) if 0/5 markers were methylated, CIMP-low (CIMP-L) if 1/5 or 2/5 markers were methylated, and CIMP-high (CIMP-H) if 3, 4 or 5/5 markers were methylated. The study cohort consisted of 14 CIMP-High, 11 CIMP-Low and 30 CIMP-Negative tumours.

### Expression analysis

Total RNA was extracted using an RNeasy MidiPrep kit (Qiagen, Hilden, Germany) and cDNA was synthesised using random hexamers and SUPERSCRIPT III (Invitrogen, Carlsbad, California). A Taqman^® ^Gene Expression Assay (part# 4331182; assay ID Hs00170261_m1 Applied Biosystems, Carlsbad CA, USA) was performed on cDNA generating a 96 bp *THBS4 *product. Gene expression was normalised to *β-actin *(*ACTB*) expression using Taqman^® ^Gene Expression Assay (part# 4331182; assay ID Hs99999903_m1 Applied Biosystems, Carlsbad CA, USA) generating a 171 bp product. The qPCR was performed in duplicate on a RotorGene3000 (Qiagen) using Absolute QPCR Mix (AB1133A; Integrated Sciences, NSW, Australia) and cycling of 40 cycles at 60°C annealing. The mathematical model described by Pffafl was used to determine the expression of *THBS4 *relative to the housekeeping gene *ACTB *[21].

### THBS4 Immunohistochemistry

THBS4 immunohistochemistry was performed on a subset of 40 patients. Briefly, fixed tumours were embedded in paraffin blocks and 0.2 μM sections were cut and mounted onto Superfrost Plus slides. They were deparaffinized in xylol and rehydrated by gradient alcohol before a 15 minute incubation with 0.5% hydrogen peroxide in phosphate buffered saline (PBS) to quench activity of endogenous peroxidases. After washes in PBS, the first containing 0.05% Triton X-100, sections were incubated in 10% goat serum and 0.01% acetylated BSA for 60 mins, then incubated overnight at 4°C in mouse anti-human THBS4 monoclonal antibody (MAB2390, R&D Systems, Minneapolis, MN) at 1:1500 in PBS/5% goat serum/1%BSA. Sections were again washed and incubated with rabbit anti-mouse Envision (Dako, Denmark) for 30 mins. The chromogenic substrate was 3,3-diaminobenzidine and sections were counterstained with Meyers' prior to dehydration and mounting of slides in Depex. Negative (no antibody) controls were included in all runs. Once the normal staining pattern for THBS4 was established, positive control normal tissue sections were also included in all runs for consistency.

### Colony formation assay

A mammalian expression construct (pcDNA3.1::*THBS4*) was designed to examine the effect of over-expressing THBS4 in CRC cell lines. Briefly, 1 μg of either pcDNA3.1(+) (Invitrogen, Carlsbad, CA, USA) or pcDNA3.1::*THBS4 *was transfected into cells using FuGENE6 (Roche Applied Science, Indianapolis, IN) at a ratio of 3:1. Three null-expressing cell lines (Lim1215, SW48 and HT29), one low-expressing cell line (SW480) and four high-expressing cell lines (DLD1, LS174T, HCT116 and RKO) were transfected in triplicate at an initial density of approximately 50%. Cells were allowed to recover for 48 hrs before application of selective media at a final concentration of 700 ng/μL G418 (GibcoBRL Life Technologies, Invitrogen, CA) for 10-14 days. At this time there were no surviving untransfected control cells, and transfected colonies were stained with 0.25% crystal violet/80% methanol. The HT29 transfection was repeated and individual colonies were expanded to confirm *THBS4 *transcript re-expression.

### Methylation Analysis

In order to determine the methylation status of the *THBS4 *promoter, qMSP was performed for 55 paired normal and tumour samples, and 13 cell lines. Briefly, 1.5 μg of DNA was modified with sodium bisulfite using the EpiTect Bisulfite kit (Qiagen, Hilden, Germany), diluted 1/8 and subjected to real time PCR in duplicate on a Rotorgene6000 (Corbett Research, QIAGEN, Germany) using Absolute QPCR Mix (AB1133A; Integrated Sciences, NSW, Australia) and cycling of 40 cycles at 60°C annealing (400 nM of F 5'-CGTTGTCGCGGAGTTTAGTA-3'; 600 nM of R 5'-ACGACGACGACGTTAACC-3'; 50 nM of Probe 5'-[DFAM]-ACCTCGATCGACGCCCGAAC[DBHQ1]). The level of methylation was determined by normalising the *THBS4 *methylation levels by the methylation levels observed for the highly methylated *ALU *gene to generate the PMR, or Percentage of Methylated Reference (400 nM of ALU-F 5'-GGTTAGGTATAGTGGTTTATATTTGTAATTT-3'; 400 nM of ALU-R 5'-ATTAACTAAACTAATCTTAAACTCCTAACCT-3'; 100 nM of ALU-Probe [6FAM]-CCTACCTTAACCTCCC-[MGBNFQ]; cycling as described for *THBS4 *qMSP above). Analysis was performed using MethyLight [22].

### Statistical Analysis

The Wilcoxon signed rank test was used to compare *THBS4 *expression as well as methylation between the normal mucosal and tumour pairs. The Kruskal Wallis test was used to test for a relationship between tumour *THBS4 *methylation and CIMP status as well as the difference in normal mucosal *THBS4 *methylation in patients with and without colorectal pathology. A non-parametric trend test was applied to confirm an ordinal gradient across multiple groups. *THBS4 *methylation within the normal colonoscopic biopsy samples was calculated at each of the four sites sampled, as well as analysed according to an average proximal (mean of cecum and transverse colon), distal (mean of sigmoid colon and rectum) and pancolorectal (mean of all four sites) result. Correlations were analyzed by the Spearman's rank (ρ) coefficient. Distal and proximal levels of methylation were analyzed by a paired t-test. All tests were performed using Stata Statistical Software, version 10 [StataCorp].

## Results

### Expression Analysis

**Figure 1:**
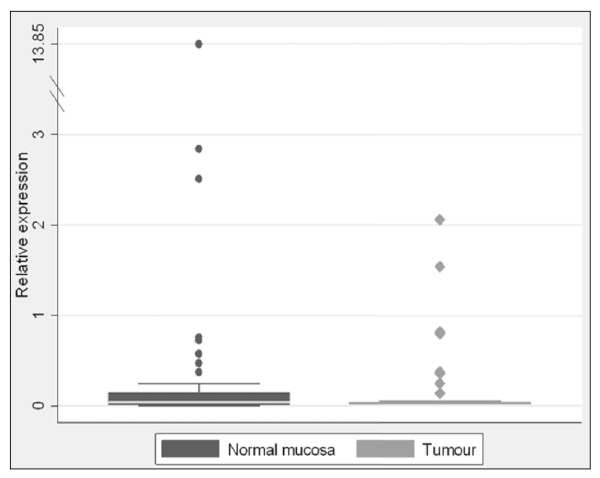
***THBS4 *expression is higher in normal mucosa than in tumour tissue**. While the relative expression of THBS4 was quite low overall, *THBS4 *expression was significantly higher in peritumoural normal mucosa than in the tumour tissue. Median expression in normal mucosa was 0.04 *vs. *0.01 for tumour tissue (p = 0.0035). Circular and diamond shaped dots represent individual data point for normal and tumours samples, respectively.

*THBS4 *expression normalised to *ACTB *was generally quite low in both peritumoural normal mucosa and in tumours (Figure 1). There was significantly higher expression of *THBS4 *in the normal mucosa compared to the tumour. Median *THBS4 *expression was 0.04 *vs. *0.01 respectively (p = 0.0035).

### THBS4 Immunohistochemistry

**Figure 2:**
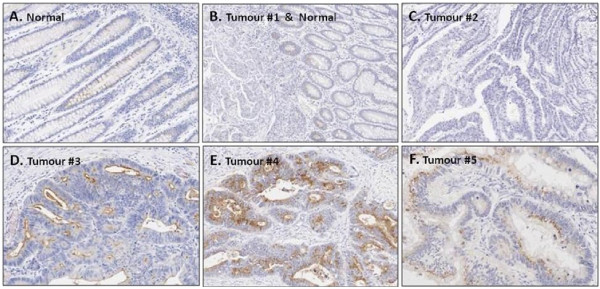
**The THBS4 protein appears to be secreted**. THBS4 protein is not expressed or is expressed at low levels in normal cells, especially towards the luminal surface. Tumour expression of *THBS4 *is heterogeneous, with generally no expression but occasional areas of strong expression and packaging in vesicular structures.

Figures 2A,B &2C show the examples of THBS4 immunohistochemical staining. In normal tissue, (Figure 2A and the right side of 2B) THBS4 protein was absent from the majority of normal epithelial cells, but was occasionally expressed in a vesicular pattern. Low levels were seen in the cytoplasm toward the luminal surface. THBS4 expression tended to be even lower in tumours compared with normal mucosa, with the majority of tumours having no detectable THBS4 expression (left side of Figure 2B, Figure 2C). In general, THBS4 protein was most likely to be seen at the luminal surface of normal crypts or tumour glands. However, there was some heterogeneity within the tumour sections and Figures 2D,E and 2F show some interesting variations to the general protein localisation in tumour sections. In Figures 2D and 2E, THBS4 protein seemed to be significantly up-regulated and actively secreted or packaged in vesicular structures (Figure 2F). These expression patterns suggest that THBS4 protein may be secreted or packaged and may give clues as to its function, although further functional studies are now required to test this hypothesis. If THBS4 protein is being packaged and secreted, it is possible that this may be one reason such low levels are detected in normal mucosa. The low frequency of detectable staining in tumour samples did not allow meaningful comparison with other molecular analyses. Immunohistochemical staining was primarily used to provide novel insight into the cellular distribution of THBS4 expression patterns.

### Colony formation assay

**Figure 3:**
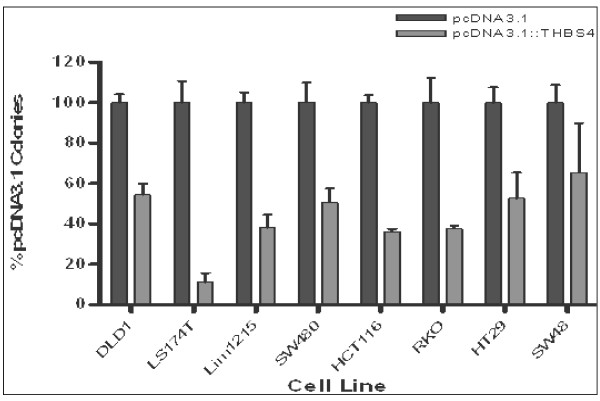
**Over-expression of THBS4 significantly decreases colony formation in CRC cell lines**. Forced over-expression of THBS4 significantly decreases colony formation in colorectal cancer cell lines regardless of their baseline expression levels. The average reduction in colony formation following THBS4 over-expression was 50-60% compared with vector-only controls. This consistent and significant reduction in colony forming ability may indicate that high levels of this protein could correlate with reduced tumour cell growth.

Colony formation was significantly repressed in all eight cell lines examined following over-expression of THBS4. We observed that both the number of colonies and their size were greatly reduced upon forced over-expression of THBS4. Figure 3 quantifies the THBS4 repression of colony formation in all cell lines tested. The reduction in colony formation ranged from 34% to 89% compared with the vector-only controls. Forced over-expression of THBS4 in multiple colorectal cancer cell lines, regardless of their basal *THBS4 *expression, consistently reduced colony forming ability. This indicates that high levels of this protein is associated with reduced tumour cell growth. To confirm that *THBS4 *transcript was introduced by transfection with this construct, HT29 was transfected and 14 individual clones were expanded. Of these, 8 showed high levels of *THBS4 *transcript whilst the remaining 6 had negligible expression levels comparable with untransfected parental HT29 cells.

### Methylation Analysis

**Figure 4:**
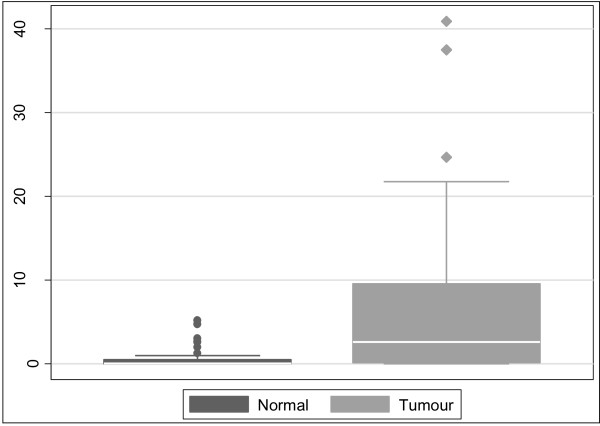
***THBS4 *methylation is significantly higher in neoplastic tissue compared to adjacent normal tissue**. *THBS4 *methylation was significantly higher in neoplastic tissues compared to the adjacent normal tissue (p < 0.0001). The median PMR in normal mucosa was 0.06 compared with a PMR of 2.6 in tumour tissue.

**Figure 5:**
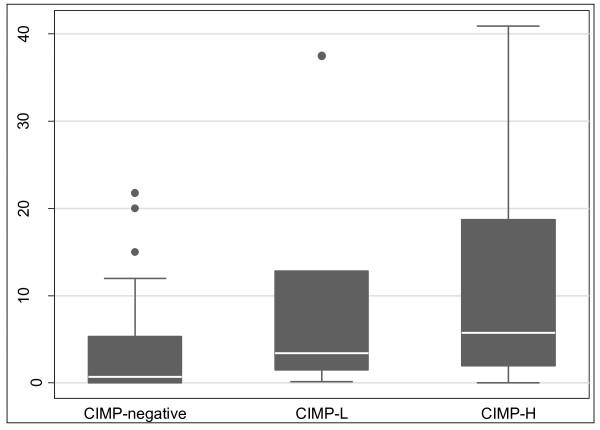
***THBS4 *methylation increases with increasing CIMP methylation**. Tumours were stratified according to the Laird classification of CIMP, separated into CIMP-neg (0/5 markers positive for methylation), CIMP-low (1/5 or 2/5 markers positive for methylation) and CIMP-high (3, 4 or 5/5 markers methylation positive) categories of methylation. *THBS4 *methylation was found to positively correlate with CIMP. The greater the number of Laird markers that were positive for CIMP, the higher the level of *THBS4 *promoter methylation. The median PMRs were 0.69 for CIMP-negative tumours, 3.4 for CIMP-L tumours, and 5.8 for CIMP-H tumours (p = 0.033).

Overall, colorectal cancers had significantly higher *THBS4 *methylation than the matched normals (Figure 4). The median PMR in normals was 0.06 *vs*. 2.6 in tumours (P < 0.0001). Tumours were classified into CIMP subgroups using the Laird marker panel [19]. When stratified by CIMP status, there was no difference in the methylation of normal mucosa between the different classes of tumours. However, *THBS4 *methylation in tumours was positively correlated with CIMP (Figure 5). Tumour PMRs showed a progressive increase with increasing level of CIMP, with the median PMR for CIMP-negative tumours being 0.69, 3.4 for CIMP-L tumours, and 5.8 for CIMP-H tumours (p = 0.033).

Using a PMR of 10 as a cut off [19], tumours were separated into *THBS4 *methylation positive (PMR ≥10) and THBS4 methylation negative (PMR < 10). *THBS4 *promoter hypermethylation (PMR ≥10) was observed in 4/30 (13.3%) CIMP-NEG tumours, 4/11 (36.4%) CIMP-L tumours, and 5/14 (35.7%) CIMP-H tumours. Thus, while the average tumour PMR correlates well with an increasing number of markers positive for CIMP, there were an equal proportion of CIMP-L and CIMP-H tumours that showed high *THBS4 *methylation.

### Correlation of Expression and Methylation of *THBS4*

In CRC cell lines, there was no significant correlation between *THBS4 *transcript expression when compared with *THBS4 *PMR (ρ = 0.42, p = 0.20) on a continuous scale (Table 1). There was also no difference in expression in the colorectal cancer cell lines tested when the PMR cut-off of ≥10 was used (p = 0.35, data not shown). However, of the 11 cell lines tested, only 3 had a PMR of < 10. Tumours were also analysed for *THBS4 *expression level. Tumours with *THBS4 *PMR ≥10 appeared to have lower *THBS4 *expression, but this difference was not significant, according to the Wilcoxon Rank-sum (Mann-Whitney) analysis (p = 0.26, data not shown). Thus, while there was a trend towards lower *THBS4 *expression in tumours with a PMR greater than or equal to 10 having decreased expression, this difference was not significant. When tumour expression levels were normalised to matched normal mucosa expression levels, 44 cancers showed reduced expression greater than 1.5 fold (average 186.5 fold down-regulation) whilst 15 cancers showed greater than 1.5 fold up-regulation (average 26.5 fold) and seven tumours did not vary in expression levels by more than 1.5 fold.

**Table 1:** *THBS4 *expression is not related to its promoter methylation in CRC cell lines

Cell Line	THBS4 Expression	THBS4 PMR
DLD1	7.24	93.68

HCT116	7.9	124.41

HT29	0	100.06

LIM1215	0	0

LIM1863	0	0

LISP1	12.92	140.51

LoVo	0.3	14.8

LS174T	7.24	2.23

RKO	6.97	224.29

SW48	0	139.72

SW480	0.89	58.6

There is no correlation between reduced *THBS4 *expression high *THBS4 *promoter methylation in CRC cell lines (ρ = 0.42, p = 0.20).

### Methylation of *THBS4 *in Normal Colonic Biopsies

**Figure 6:**
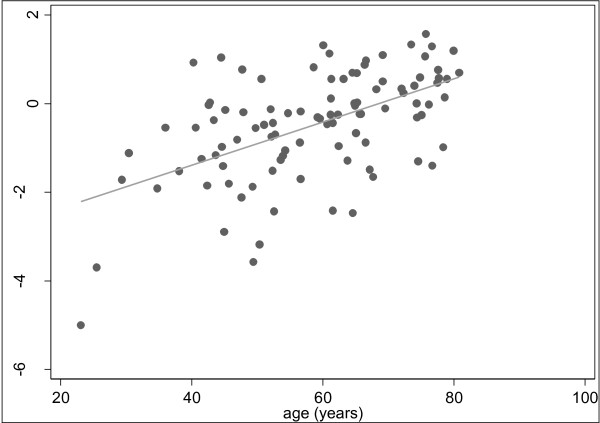
***THBS4 *methylation is associated with ageing in normal colorectal mucosa**. A strong correlation was observed between *THBS4 *methylation (presented as the log transformed pancolorectal result) and age in the normal mucosa from the colonoscopy study (ρ = 0.50, P < 0.0001). Because of this strong relationship with aging, we have postulated that *THBS4 *is a Type A (age-related) marker of methylation.

*THBS4 *methylation was detected within the normal mucosa in all 99 of the colonoscopy patients with similar levels of methylation within the proximal and distal colorectum (median PMR 0.66 *vs. *0.65, respectively, p = 0.66). Additionally, there were no differences in *THBS4 *methylation between the sexes, either at individual sites or in terms of pancolorectal *THBS4 *methylation. There was however, a strong and direct correlation between patient age and pancolorectal *THBS4 *methylation (ρ = 0.50, p < 0.0001, Figure 6).

**Figure 7:**
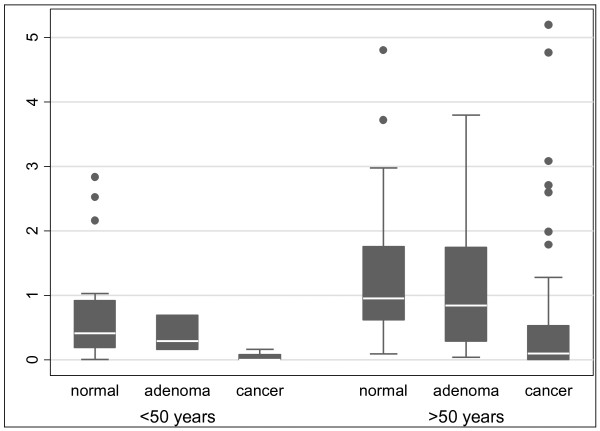
**Relationship of *THBS4 *methylation within the normal mucosa to pathology present elsewhere within the colorectum**. *THBS4 *methylation within the normal mucosa with respect to the pathology present elsewhere within the colorectum was stratified by age (< 50 years, p = 0.018 and > 50 years, p = 0.0001). There was a significant trend evident in both groups p = 0.007 and p < 0.001 for young colorectum (< 50 yrs) and aged colorectum (> 50 yrs) respectively, indicating a significant inverse relationship between adenomatous pathology and *THBS4 *methylation. The lowest levels of *THBS4 *promoter methylation were associated with the most advanced pathology in both young and aged colorectal mucosa.

*THBS4 *methylation was lower in the background mucosa of patients with colorectal adenomas compared to those with normal colonoscopies. Inclusion of the peritumoural normal mucosal samples and stratification by age, revealed a consistent and inverse association between adenomatous pathology and *THBS4 *methylation (Figure 7). Importantly, *THBS4 *methylation within the normal colorectal field showed an impressive and inverse biological gradient with the presence of colorectal pathology, with the lowest levels of *THBS4 *methylation within the mucosal field being associated with the most advanced pathology (Figure 7). This suggested that *THBS4 *methylation in the normal mucosa was not directly implicated in promoting colorectal neoplasia and could in fact be directly or indirectly protective. This is supported by our DNA methylation data in other "type A" genes [23].

## Discussion

*Thrombospondin-4 *is a putative tumour-suppressor gene that plays an integral role in mediating cellular processes such as cell attachment and migration [4]. Classes of tumour suppressor genes include those involved with DNA repair, cell growth, cell cycling, cell adhesion, cell migration, transcriptional regulation and apoptosis [24]. These may be inactivated through gene mutation, chromosomal deletion or methylation [25]. Tumour suppressor genes encode proteins which, upon loss of function, such as through epigenetic silencing, leads to a selective growth advantage for neoplastic cells. *THBS4 *has shown to be methylated in several tumour types, and this methylation is associated with transcriptional down-regulation [6,8,9].

Thrombospondin-4 is expressed at low levels in normal colonocytes, especially in the cytoplasm towards the luminal surface. Expression in tumours tends to be even lower than the levels observed in normal mucosa. Immunohistochemical localisation of THBS4 confirmed the absence of protein expression in the majority of tumours. However, some cancer cells within particular regions of the individual tumours have higher expression. A subset of tumours did express THBS4 and evidently secrete the protein, observed as more intense staining of vesicular structures towards the luminal surface. Indeed, THBS4 can been readily collected from culture supernatants of cells expressing THBS4 constructs [26], or display prominent ER and Golgi labelling [3], indicating that it is a secreted protein. While the secretion of the THBS4 protein may account for the low levels of staining observed, qPCR also demonstrated that gene expression is generally quite low. *Thrombospondin-4 *expression by qPCR is significantly higher in normal tissues than in matched tumour samples, supporting the notion that loss of THBS4 provides a selective growth advantage to cancer cells.

The forced over-expression of THBS4 in cancer cell lines significantly suppresses their colony forming abilities and growth, regardless of their baseline THBS4 expression levels. This was demonstrated *in vitro *for all 8 CRC cell lines tested, each with varying basal levels of *THBS4 *expression. A consistent and significant growth suppression of 50-60% was observed following the forced over-expression of THBS4. This demonstrates that a large amount of THBS4 protein correlates with reduced cell growth, and strengthens the evidence supporting its role as a tumour suppressor gene. Further experiments are now required to investigate the mechanism of this reduced growth, which may be a direct result of THBS4-mediated tumour suppression or toxicity due to higher than physiologically normal levels of THBS4 protein.

The Laird methylation marker panel used to determine CIMP status of colorectal cancers consists of the CACNA1G, IGF2, NEUROG1, RUNX3 and SOCS1 genes [19]. *THBS4 *methylation in the tumours, as quantitatively measured by MethyLight, significantly increased with the increasing CIMP status of tumours. The greater the number of CIMP markers that were positive for methylation (PMR ≥10), the higher the level of *THBS4 *methylation, although the same proportion of CIMP-L and CIMP-H tumours demonstrated high *THBS4 *methylation. This suggests that *THBS4 *methylation may have a role in progression of CIMP positive CRC, regardless of level of CIMP.

We postulated that methylation of the *THBS4 *promoter region would cause reduction in gene expression level. However, although normal mucosa has significantly higher expression than tumour tissue and significantly lower methylation than tumour tissue, there was no statistically significantly correlation between high promoter methylation and reduced gene expression. Reduced *THBS4 *expression levels did not correlate with a *THBS4 *PMR of 10 or greater in either cancers or cell lines. Nevertheless, in another study, methyltransferase inhibition by 5-azadeoxycytidine in SW48 cells resulted in reactivation of silenced *THBS4 *[6]. Thus, it appears that other factors may control expression of this gene, and that the moderate levels of *THBS4 *methylation observed in our study are not enough to significantly inhibit gene expression. It is also possible that other CpG sites within the *THBS4 *gene promoter that were not examined in this study are important for regulating gene expression

The genes methylated in CRC may be characterized as "type A" (*Age-related*) genes and "type C" (*Cancer-specific*) genes [27]. Generally, "type A" genes are methylated in both normal and tumour tissue and their degree of methylation is proportional to the age of the normal tissue [14,27,28]. The methylation of "type C" genes, however, is more specific for neoplastic tissue [14,27-30]. From this study, *THBS4 *behaved as a "type A" gene. *THBS4 *exhibited methylation in both normal and tumour tissue and displayed a positive correlation with age. Furthermore, as demonstrated in a recent study, [23]*THBS4 *behaved as a "type A" marker with respect to its association with neoplasia found elsewhere within the field. Methylation of the *THBS4 *promoter was highest in normal mucosa in patients with normal colonoscopies, and declined progressively as more advanced pathology was evident (Figure 7).

## Conclusions

*THBS4 *may act as a tumour suppressor gene, evidenced by the dramatic repression of colony formation upon forced over-expression in CRC cell lines and its generally lower expression in cancers compared to normal mucosa. *THBS4 *methylation is higher in cancers that also exhibit the CIMP phenotype, which is demonstrated by the strong correlation with the Laird panel of methylation markers. However, it appears that methylation has not reached a critical level where it alters *THBS4 *gene expression, or that *THBS4 *expression is regulated by factors in addition to promoter hypermethylation. *THBS4 *promoter methylation occurs in a manner representative of a Type A gene, which is methylated in normal aged colon as part of the aging process. Additional studies are required to identify other genes that are involved in the regulation of *THSB4*.

## List of Abbreviations

THBS4: Thrombospondin-4; CIMP: CpG Island Methylator Phenotype; CRC: colorectal cancer

## Competing interests

The authors declare that they have no competing interests.

## Authors' contributions

SG contributed to the acquisition of data (expression, methylation and cell line studies), analysis and interpretation of data and drafting of the manuscript.

JC contributed to the acquisition of data (expression and methylation studies), analysis and interpretation of data. KI contributed to the acquisition of data (expression studies), analysis and interpretation of data and drafting of the manuscript. SC contributed to the acquisition of data (expression studies), analysis and interpretation of data. IR contributed to the acquisition of data (CIMP classification) and analysis and interpretation of data. RB contributed to the acquisition of data (methylation in normal mucosa) and analysis and interpretation of data. KS contributed to the acquisition of data (cell line studies), study concept and design. GB contributed to the acquisition of data (cell line studies), study concept and design. DW contributed to the acquisition of data (methylation in normal mucosa), analysis and interpretation of data, statistical analysis and drafting and critical revision of the manuscript. BL contributed to the study concept and design and critical revision of the manuscript. VW contributed to the study concept and design, acquisition of data (expression and methylation studies, immunohistochemistry and cell line studies), drafting and critical revision of the manuscript. All authors have reviewed and approved the final manuscript.

## Pre-publication history

The pre-publication history for this paper can be accessed here:

http://www.biomedcentral.com/1471-2407/10/494/prepub
